# Scriptaid Treatment Decreases DNA Methyltransferase 1 Expression by Induction of MicroRNA-152 Expression in Porcine Somatic Cell Nuclear Transfer Embryos

**DOI:** 10.1371/journal.pone.0134567

**Published:** 2015-08-11

**Authors:** Shuang Liang, Ming-Hui Zhao, Jeong-woo Choi, Nam-Hyung Kim, Xiang-Shun Cui

**Affiliations:** 1 Department of Animal Science, Chungbuk National University, Cheongju, Chungbuk, 361–763, Republic of Korea; 2 Brain Korea 21 center for Bio-Resource Development, Cheongju, Chungbuk, 361–763, Republic of Korea; Institute of Zoology, Chinese Academy of Sciences, CHINA

## Abstract

Abnormal epigenetic reprogramming of donor nuclei after somatic cell nuclear transfer (SCNT) is thought to be the main cause of low cloning efficiencies. A growing body of evidence has demonstrated a positive role of Scriptaid, a histone deacetylase inhibitor (HDACi) that belongs to an existing class of hydroxamic acid-containing HDACis, on the development competence of cloned embryos in many species. The present study investigated the effects of Scriptaid on the development of porcine SCNT embryos *in vitro* and its mechanism. Treatment with 300 or 500 nM Scriptaid for 20 h after activation significantly increased the percentage of SCNT embryos that developed to the blastocyst stage and the total number of cells per blastocyst and significantly decreased the percentage of apoptotic cells in blastocysts. Scriptaid treatment significantly increased the level of histone H3 acetylated at K9 and the conversion of 5-methylcytosine into 5-hydroxymethylcytosine and significantly decreased the level of histone H3 trimethylated at K9 at the pronuclear stage. As a potential mechanism for the DNA methylation changes, our results showed that the expression of DNA methyltransferase 1 was frequently down-regulated in Scriptaid-treated embryos in comparison with untreated embryos and was inversely correlated to endogenous microRNA-152 (miR-152). Taken together, these findings illustrated a crucial functional crosstalk between miR-152 and DNMT1. Meanwhile, mRNA and protein levels of POU5F1 and CDX2 were increased in Scriptaid-treated embryos. mRNA levels of *Caspase3*, and *Bax* were significantly decreased and that of *Bcl-xL* was significantly increased in Scriptaid-treated embryos. In conclusion, these observations would contribute to uncover the nuclear reprogramming mechanisms underlying the effects of Scriptaid on the improvement of porcine SCNT embryos.

## Introduction

Although several mammalian species have been successfully cloned using somatic cell nuclear transfer (SCNT) technology [1–3], its success rate remains extremely low, especially in pigs [4]. This low efficiency is mostly attributed to defective epigenetic reprogramming, including genomic methylation. Histone tail modifications may result in abnormal epigenetic modification and gene expression in SCNT embryos [5–8]. Hence, epigenetic modifications might be key to improving the success of cloning. Recent studies showed that specific histone deacetylase inhibitors (HDACis) enhance somatic cell genomic reprogramming and repair epigenetic abnormalities, such as acetylation of histone H3 at K9 (H3-acK9)[9].

HDACis induce specific changes in gene expression and influence a variety of processes, including growth arrest, differentiation, cytotoxicity, and induction of apoptosis [10]. HDACis may also damage embryos [11]. Thus, avoidance of the side-effects of HDACi treatment is probably important to improve the cloning efficiency and to modify aberrant genomic reprogramming.

Histone deacetylases (HDACs) are divided into five categories: class I (HDAC 1–3 and 8), class IIa (HDAC 4, 5, 7, and 9), class IIb (HDAC 6 and 10), class III (SIRT 1–7), and class IV (HDAC 11) [12]. Scriptaid can inhibit classes I and IIa/b HDACs [12, 13], belongs to an existing class of hydroxamic acid-containing HDACis, is less toxic than other HDACis, and causes increases in the global acetylation of histones, transcriptional activity, and protein expression [14].

HDACs and microRNAs (miRNAs) have a complex relationship that is not fully understood, but may be of critical importance. miRNAs can regulate HDACs and influence histone acetylation, and HDACs can regulate miRNA expression. Thus, a careful balance between HDACs and miRNAs is important to maintain their appropriate levels in the cell [15]. HDACis can alter the expression profiles of miRNAs in some cells [16, 17]. In our previous study, aberrant epigenetic reprogramming of imprinted mir-127 was observed in cloned mouse embryos [18]. Thus, understanding of how HDACs and miRNAs influence each other and affect biological pathways is of great interest.

Scriptaid treatment significantly improves the development of cloned pig embryos *in vitro* and *in vivo* and restores the correct expression of specific aberrantly expressed genes [19]. Furthermore, Scriptaid treatment of porcine SCNT embryos improves the cloning efficiency and nuclear reprogramming of inbred miniature pigs [19–21]. Similar results were obtained with embryos of several mammalian species [22, 23]. These data indicate that Scriptaid is a promising candidate to improve the *in vitro* development of porcine embryos; however, the mechanism underlying its effects needs to be elucidated.

In general, embryos produced *in vitro* contain fewer cells and have a higher apoptotic index than embryos produced *in vivo* [24]; this is particularly true of cloned embryos [25]. An increased apoptotic rate might contribute to embryo mortality or fetal anomalies that can trigger early abortions [26]. Thus, further studies are required to evaluate factors that affect the quality of SCNT embryos as well as apoptosis in these embryos.

This study investigated the mechanism by which treatment of Scriptaid on the reprogramming of somatic nuclei following SCNT and on the in vitro development of SCNT embryos.

## Materials and Methods

This research was carried out in strict accordance with the recommendations in the Guide for the Care and Use of Laboratory Animals of the National Veterinary and Quarantine Service. The protocol was approved by the Committee on the Ethics of Animal Experiments of the Chungbuk National University (Permit Number: CBNUA-681-14-01). All chemicals used in this study were purchased from Sigma Chemical Company (Sigma, St. Louis, MO, USA), unless otherwise indicated. Scriptaid was dissolved in dimethyl sulfoxide, prepared as a 2000× stock solution of 500 μM, aliquoted, and stored in the dark at −20°C. The stock solution was added to porcine zygote medium (PZM)-5 culture media at various concentrations using serial dilution.

### Collection of porcine oocytes and *in vitro* maturation

Ovaries from pre-pubertal gilts were collected from a local slaughterhouse (Farm story dodarm B&F, Umsung, Chungbuk, Korea) and transported to the laboratory at 37°C in saline supplemented with 75 mg/ml penicillin G and 50 mg/ml streptomycin sulfate. Follicles that were 3–6 mm in diameter were aspirated. Cumulus-oocyte complexes (COCs) that were surrounded by a minimum of three cumulus cells were selected for culture [27]. In brief, the COCs were washed three times in TL-HEPES supplemented with 0.1% polyvinyl alcohol (PVA, w/v) and 0.05 g/L gentamycin. Then the COCs were washed three times in maturation medium (TCM-199 supplemented with 0.1 g/L sodium pyruvate, 0.6 mM L-cysteine, 10 ng/mL epidermal growth factor, 10% porcine follicular fluid, 10 IU/mL luteinizing hormone, and 10 IU/mL follicle-stimulating hormone) and were then transferred to maturation medium. Maturation was performed by culturing approximately 50 COCs in 500 μL of maturation medium in 4-well dishes. The medium was covered with mineral oil and the plates were incubated at 38.5°C in a humidified atmosphere of 5% CO_2_ for 44 h. The pH and osmolality of the maturation medium were 7.35 and 290 mmol/kg, respectively.

### SCNT procedure

After 44 h of *in vitro* maturation, cumulus cells were removed from the oocyte by gentle pipetting in TL-HEPES supplemented with 1 mg/mL hyaluronidase and 0.1% PVA, collected in a 1.5-ml Eppendorf tube, washed by centrifugation, maintained at 4°C, and used as donor cells.

For enucleation, only oocytes with an excellent morphology and that had extruded the first polar body, were used for SCNT. Denuded oocytes were incubated for 5 min in manipulation medium (calcium-free TL-HEPES supplemented with 0.1% PVA) containing 5 μg/mL Hoechst 33342, washed twice with fresh manipulation medium, and transferred to a drop of manipulation medium containing 5 μg/mL cytochalasin B (CB). Oocytes were enucleated by aspirating the polar body and MII chromosomes in a small amount (<15% of the oocyte volume) of cytoplasm using a 25-μm beveled glass pipette (Humagen, Charlottesville, VA, USA).

After enucleation using a fine injecting pipette, a single donor cell was inserted into the perivitelline space of the enucleated oocyte. Donor cell-oocyte complexes were equilibrated with 280 mM mannitol solution (pH 7.2) containing 0.15 mM MgSO_4_, 0.01% PVA (w/v), and 0.5 mM HEPES for 2–3 min and transferred to a fusion chamber containing two electrodes overlaid with 280 mM mannitol solution. Membrane fusion was induced by applying an alternating current field of 2 V cycling at 1 MHz for 2 sec, followed by a 20-μsec direct current (DC) pulse at 2 kV/cm using a cell fusion generator (LF201; Nepa Gene, Chiba, Japan). Following fusion, the reconstructed embryos were placed in bicarbonate-buffered PZM-5 containing 0.4 mg/mL bovine serum albumin (BSA) for 1 h prior to activation.

### Activation and in vitro culture

Reconstructed embryos were activated by two DC pulses of 120 V for 60 μsec in 297 mM mannitol (pH 7.2) containing 0.1 mM CaCl_2_, 0.05 mM MgSO_4_, 0.01% PVA (w/v), and 0.5 mM HEPES. Following activation, the reconstructed embryos were cultured in bicarbonate-buffered PZM-5 containing 0.4 mg/mL BSA and 7.5 μg/mL CB for 3 h to suppress extrusion of the pseudo-second polar body. Following culture, the reconstructed embryos were thoroughly washed and cultured in bicarbonate-buffered PZM-5 supplemented with 0.4 mg/mL BSA in 4-well dishes for 7 days at 38.5°C in 5% CO_2_ in air without changing the medium. The development of the reconstructed embryos into blastocysts was examined on day 7 after activation.

### Real-time reverse transcription-polymerase chain reaction (RT-PCR) with SYBR green for mRNA analysis

mRNAs from SCNT embryos were isolated using a Dynabeads mRNA Direct Kit (Dynal Asa, Oslo, Norway), according to the manufacturer’s instruction. First-strand cDNA was synthesized by RT of mRNA using the Oligo(dT)_12-18_ primer and SuperScript TM III Reverse Transcriptase (Invitrogen Co., Grand Island, NY). Real-time RT-PCR using the CFX96 Touch Real-time RT-PCR Detection System (Bio-Rad) was performed in a final reaction volume of 20 μL with SYBR Green, a fluorophore that binds all double-strand DNA. The PCR conditions were as follows: 5 min at 95°C followed by 45 cycles of 10 sec at 95°C, 10 sec at 60°C, and 15 sec at 72°C. Finally, gene expression was quantified using the 2-ddCt method, with normalization to the mRNA expression of porcine ribosomal protein L19 (*Rpl19)*. The primers used to amplify each gene are listed in Table 1. Each experiment was repeated at least three times, with five embryos per repeat.

**Table 1:** Primers used for real-time RT-PCR.

Gene	Primer sequences (5'-3')	Annealing temperature (°C)	Product size (bp)
*Bcl-xL*	F: CTTACCTGAATGACCACCTAGAGC	60	182
	R: CCGACTGAAGAGCGAACCC		
*Bax*	F: CGGGACACGGAGGAGGTTT	60	189
	R: CGAGTCGTATCGTCGGTTG		
*Casp3*	F: ACTGTGGGATTGAGACGG	55	110
	R: GGAATAGTAACGAGGTGCTG		
*Dnmt1*	F: GGCAGACCACCATCACATC	55	165
	R: GGAGCAGTCCGGCAACT		
*Dnmt3a*	F: GGACAAGAATGCCACCAAATCA	60	196
	R: CTTGCCGTCTCCGAACCA		
*Dnmt3b*	F: GGGTGGAAAGACACGGGAT	60	243
	R: TAGGAGCGTAGAAGCAAGGAA		
*Pou5f1*	F:GCTCACTTTGGGGGTTCTCT	60	228
	R:TTGCCTCTACTCGGTTCTC		
*Cdx2*	F: GCAAAGGAAAGGAAAATCAACAA	60	120
	R: GGGCTCTGGGACGCTTCT		
*Rpl19*	F: GCTTGCCTCCAGTGTCCTC	60	179
	R: GGCGTTGGCGATTTCAT		

### Real-time RT-PCR with TaqMan for microRNA analysis

All primers, including those used for specific miRNA and cDNA synthesis and PCR amplification, and the kit used for miRNA analyses were purchased from Applied Biosystems (Bedford, MA, USA). For relative quantification of the expression of mir-29b, mir-148a, mir-152, and U6 snoRNA, RT reactions were set up containing 5 μL RNA, 3 μL stem-loop RT primer, 1× RT buffer, 0.25 mM of each dNTP, 3.33 U/mL MultiScribe reverse transcriptase, and 0.25 U/mL RNase inhibitor. The 15-μL reactions were incubated for 30 min at 16°C, followed by 30 min at 42°C and 5 min at 85°C, and were then held at 4°C to convert miRNA into cDNA. Real-time RT-PCR was performed in 20-μL reactions that included 4 μL RT product, 1× TaqMan Universal PCR Master Mix, and 1 μL of 20× real-time solution containing TaqMan probe and primers. The amplification parameters used for real-time RT-PCR were as set out in the manufacturer’s protocol. miRNA expression was quantified using the same method as that described for mRNA expression, with U6 snoRNA used as an internal control. Each experiment was repeated at least three times, with five embryos per repeat.

### Immunostaining and quantification of fluorescence intensity

Scriptaid-treated and non-treated embryos were collected at the pronuclear stage. Embryos were fixed for 30 min in 3.7% (w/v) paraformaldehyde, permeabilized for 1 hour with 0.2% Triton X-100 prepared in PBS-PVA, and then blocked for 30 min at room temperature in 1% BSA prepared in PBS-PVA. Next, the embryos were incubated with a rabbit polyclonal antibody against H3-acK9 (Abcam, ab10812, Cambridge, UK) diluted 1:100, a rabbit polyclonal antibody against Dnmt1 (Santa Cruz Biotechnology, sc-20701, CA, USA), a rabbit polyclonal antibody against H3-m3K9 (Abcam, ab8898, Cambridge, UK), a rabbit polyclonal antibody against 5mc and 5hmc (Abcam, ab124936 and ab106918, Cambridge, UK), a rabbit polyclonal antibody against POU5F1 (Santa Cruz Biotechnology, sc-9081, CA, USA), or a rabbit polyclonal antibody against CDX2 (Santa Cruz Biotechnology, sc-134468, CA, USA), which were all diluted 1:200 overnight at 4°C. After washing extensively with 0.2% Tween-20 prepared in PBS, embryos were incubated with Alexa Fluor-594-labeled goat anti-rabbit IgG (Invitrogen, Carlsbad, CA, USA) diluted 1:200 for 1 h at 37°C. The embryos were mounted onto slides using mounting medium containing 10 μg/mL Hoechst 33342 to stain DNA for 5 min. Images were captured using a laser scanning confocal microscope (Zeiss LSM 510 and 710 META, Oberkochen, Germany) and the appropriate excitation wavelength and exposure time. Images of embryos were analyzed using Image Pro Plus 6.0 software (Media Cybernetics, USA). In brief, target signal was recognized as (region of interest (ROI) by the software and fluorescence intensity per pixel were measured by the software automatically. The mean fluorescence intensities of H3-acK9, H3-m3K9, 5mc, 5hmc and Dnmt1 labeling were calculated and compared among the groups. Each experiment was repeated at least three times, with 10–15 embryos per repeat.

### Counting the number of nuclei per blastocyst

Blastocysts at day 7 were collected from each group and fixed in 3.7% paraformaldehyde prepared in PBS-PVA for 30 min at room temperature. The embryos were mounted onto slides using mounting medium containing 10 μg/mL of Hoechst 33342. After rinsing with PBS-PVA, stained blastocysts were mounted onto glass slides beneath a coverslip, and the number of cells per blastocyst was examined under an inverted epifluorescence microscope (Nikon Corp., Tokyo, Japan).

### Terminal deoxynucleotidyl transferase-mediated dUTP nick-end labeling (TUNEL) assay

Blastocysts were washed three times in PBS (pH 7.4) containing 1 mg/mL polyvinylpyrolidone (PBS/PVP) and then fixed in 3.7% paraformaldehyde prepared in PBS for 1 hour at room temperature. After fixation, the embryos were washed in PBS/PVP and permeabilized by incubation in 0.3% Triton X-100 for 1 hour at room temperature. Thereafter, the embryos were washed twice in PBS/PVP and incubated with fluorescein-conjugated dUTP and the terminal deoxynucleotidyl transferase enzyme (In Situ Cell Death Detection Kit, Roche; Mannheim, Germany) in the dark for 1 hour at 37°C. After being incubated with 10 μg/mL Hoechst 33342 and 50 mg/mL RNase A for 1 hour at 37°C to label all nuclei, embryos were washed in PBS-PVA, mounted with slight coverslip compression, and examined using laser scanning confocal microscope (Zeiss LSM 510 and 710 META, Oberkochen, Germany).

### Experimental design

#### In Experiment 1

Reconstructed embryos treated with various concentrations of Scriptaid were examined to determine the optimal concentration. After activation, reconstructed embryos were cultured in PZM-5 medium supplemented with 0, 100, 300, or 500 nM Scriptaid for 20 h, and were then transferred to medium lacking Scriptaid. The percentage of embryos that developed to the blastocyst stage and the total number of cells per blastocyst were examined in each group.

**Fig 1:**
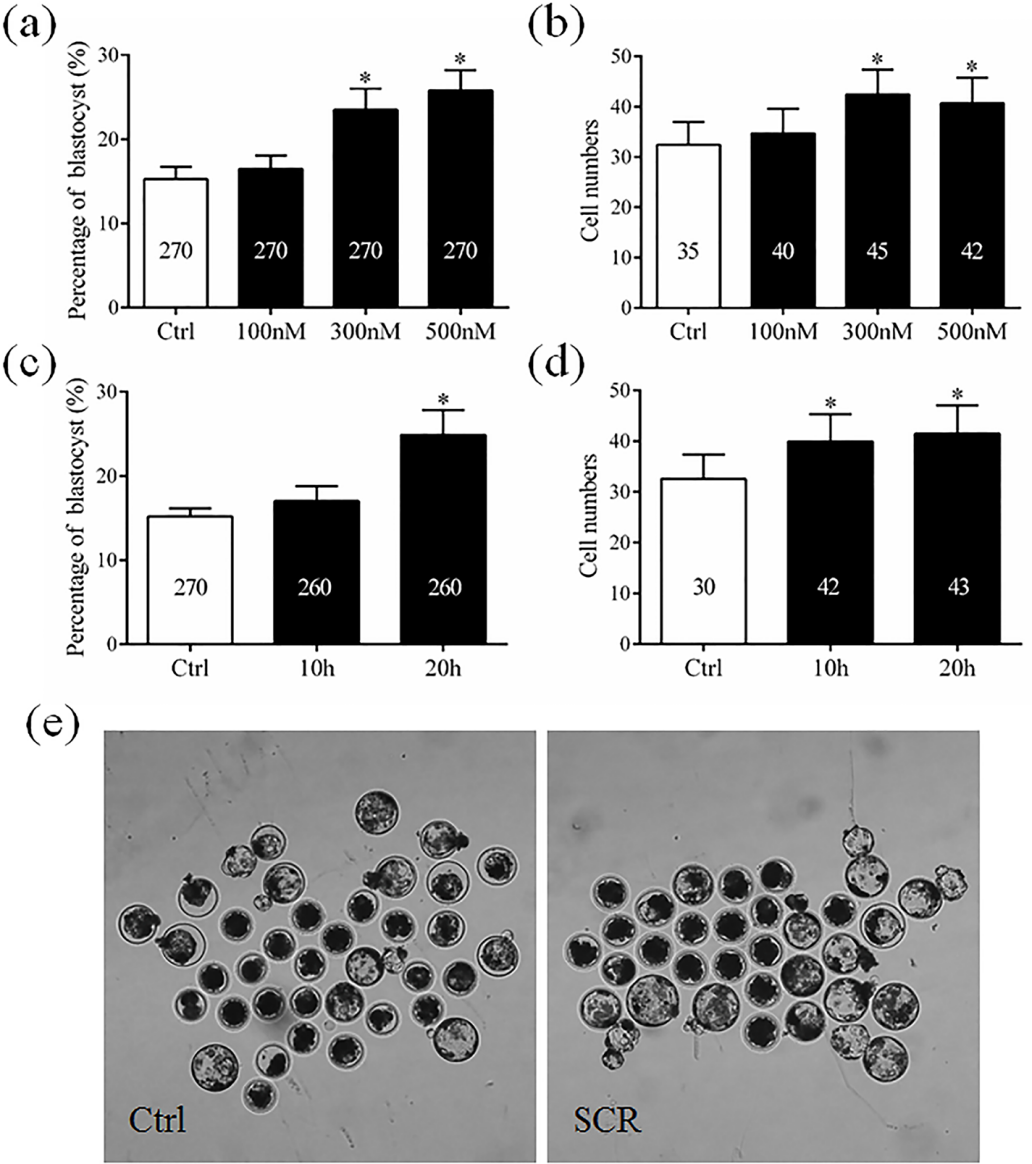
Effect of treatment with Scriptaid at various concentrations (a and b) and for various amounts of time (c, d, and e) on the development of porcine somatic cell nuclear transfer embryos to the blastocyst stage. The numbers of embryos examined in each experimental group are shown in the bars. * indicates P<0.05 compared with the control group. Values are the mean (± standard deviation of the mean) of four independent experiments. In E, embryos were not treated or were treated with 300 nM Scriptaid for 20 h. Ctrl: no treatment; SCR: Scriptaid treatment. Magnification, × 100.

From this, 300 nM was selected as the optimal concentration of Scriptaid (Fig 1A and 1B). Reconstructed embryos were cultured with 300 nM Scriptaid for various amounts of time to determine the optimal duration of treatment. After activation, reconstructed embryos were cultured in medium supplemented with 300 nM Scriptaid for 0, 10, or 20 h, and were then transferred to medium lacking Scriptaid. The percentage of embryos that developed to the blastocyst stage and the total number of cells per blastocyst were examined in each group.

#### Experiment 2

Examined the effects of Scriptaid treatment on the number of apoptotic cells, the total number of cells, and the expression of apoptosis-related genes in blastocysts. *In vitro*-cultured embryos obtained as described in Experiment 1 were harvested at the blastocyst stage on day 7 and subjected to the TUNEL assay. The total number of nuclei per blastocyst was also counted. Blastocysts were washed in PBS and stored at −80°C until RT-PCR analysis.

#### In Experiment 3

To determine the levels of histone acetylation, histone methylation, and DNA methylation in SCNT embryos treated with Scriptaid, reconstructed embryos treated with or without 300 nM Scriptaid for 20 h were collected at the pronuclear stage (15 h). Thereafter, the fluorescence intensities of labeling for the epigenetic markers H3-acK9, H3-m3K9, 5hmc, 5mc and *Dnmt1* were determined.

#### In Experiment 4

To investigate the effects of Scriptaid treatment on the relationship between DNA methylation and miRNA expression, the expression levels of DNA methyltransferases (*Dnmt1*, *Dnmt3a*, and *Dnmt3b*) and miRNAs (mir-29b, mir-148a, and mir-152) were determined in Scriptaid-treated and non-treated SCNT embryos.

#### In Experiment 5

To investigate the effects of Scriptaid treatment on mRNA and protein expression during early embryonic development, the expression levels of development-related genes and proteins (*Pou5f1* and *Cdx2*) were determined in Scriptaid-treated and non-treated SCNT embryos.

### Statistical analysis

Each experiment was repeated at least three times. All embryos were randomly allocated to a treatment group. Data were analyzed with a one-way analysis of variance and Tukey’s least significant test using GraphPad Prism 6 software. p<0.05 was considered significant.

## Results

### Effects of treatment with Scriptaid at various concentrations and for various amounts of time on the in vitro development of SCNT embryos

After activation, SCNT embryos were treated with various concentrations of Scriptaid (0, 100, 300, and 500 nM) for 20 h and their *in vitro* development was examined. The percentage of embryos that developed to the blastocyst stage was significantly higher among embryos treated with 300 or 500 nM Scriptaid than among those treated with 100 nM Scriptaid and non-treated embryos (300 nM, 23.50%; 500 nM, 25.78%; 100 nM, 16.48%; non-treated, 15.26%; Fig 1A). However, the percentage of embryos that developed to the blastocyst stage did not significantly differ between those treated with 300 nM Scriptaid and those treated with 500 nM Scriptaid. Similar results were observed for the total number of cells per blastocyst (Fig 1B), which was significantly higher among embryos treated with 300 nM or 500 nM Scriptaid than among those treated with 100 nM Scriptaid and non-treated embryos.

Based on these results, 300 nM was chosen as the optimal concentration of Scriptaid. Next, the effects of treatment with 300 nM Scriptaid for various amounts of time (0, 10, and 20 h) on the *in vitro* development of SCNT embryos were analyzed. The percentage of SCNT embryos that developed to the blastocyst stage was significantly higher among those treated with Scriptaid for 20 h (24.90%) than among those treated with Scriptaid for 0 h (15.19%) or 10 h (17.02%) (Fig 1C and 1E). The total number of cells per blastocyst was significantly higher among Scriptaid-treated embryos (10 or 20 h) than among non-treated embryos (Fig 1D).

### Effects of Scriptaid treatment on apoptosis-related gene expression in the blastocysts

**Fig 2:**
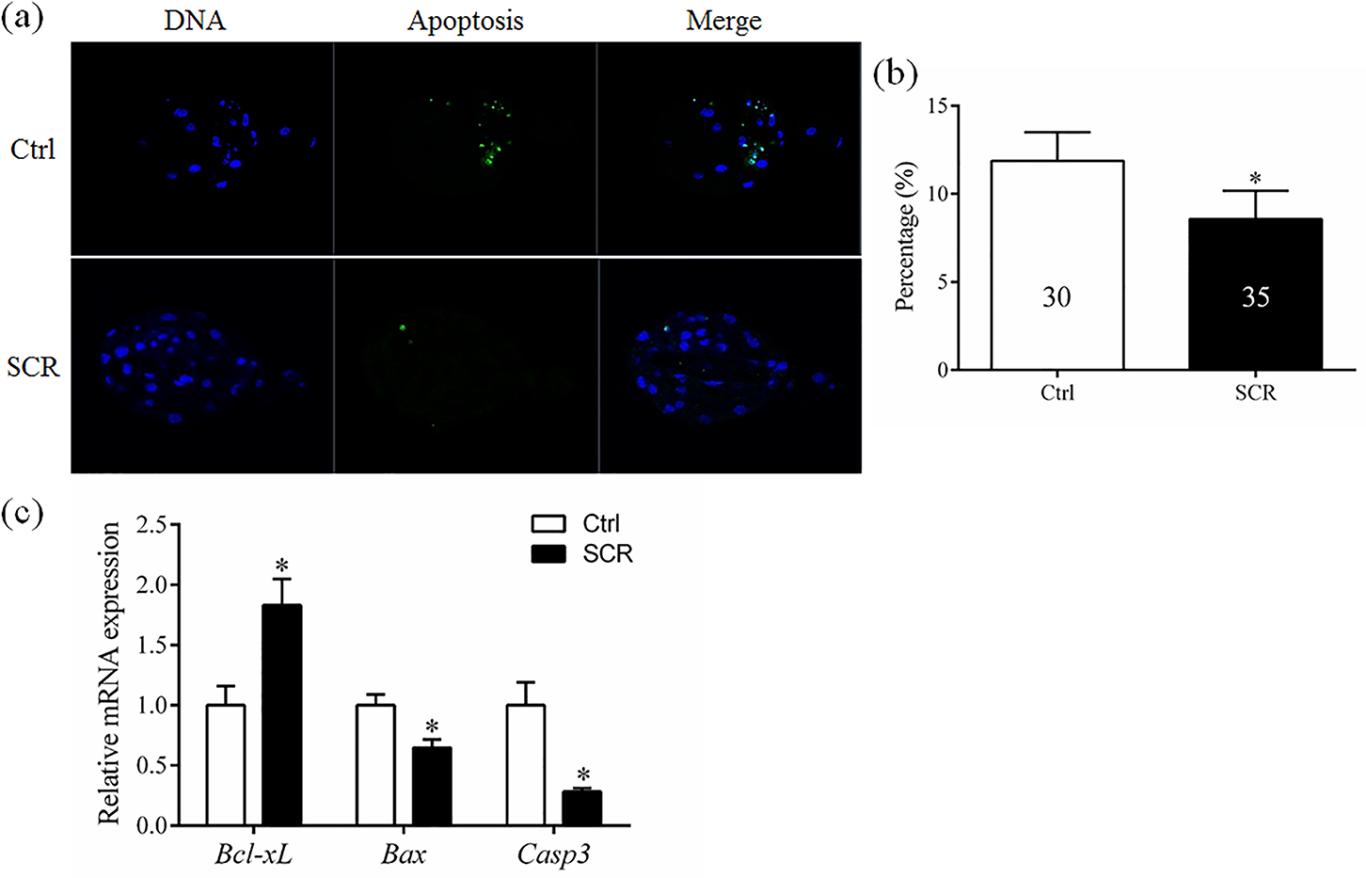
Laser scanning confocal microscopy images of nuclei and fragmented DNA (×400, a and b) and relative mRNA expression levels of *Bcl-xL*, *Bax*, and *Caspase3* (*Cas3*) (c) in porcine blastocysts after 7 days of *in vitro* culture, with or without Scriptaid treatment. The numbers of embryos examined in each experimental group are shown in the bars. * indicates P<0.05 compared with the control group. Values are the mean (± standard deviation of the mean) of four independent experiments. Ctrl: no treatment; SCR: Scriptaid treatment.

DNA fragments generated by apoptotic nicking of genomic DNA were measured in individual embryos using the TUNEL assay. The percentage of apoptotic cells in blastocysts was significantly lower in Scriptaid-treated embryos than in non-treated embryos (Fig 2A and 2B). The effects of Scriptaid treatment on expression of the apoptosis-related genes *B-cell lymphoma-extra large (Bcl-Xl)*, *Bcl-2-associated X protein (Bax)*, and *Caspase3* (*Casp3*) were determined in SCNT embryos at the blastocyst stage. In comparison with non-treated blastocysts, mRNA expression of *Cas3* and *Bax* was significantly lower and mRNA expression of *Bcl-xL* was significantly higher in Scriptaid-treated blastocysts (Fig 2C).

### Effects of Scriptaid treatment on histone acetylation, histone methylation, and DNA modification

**Fig 3:**
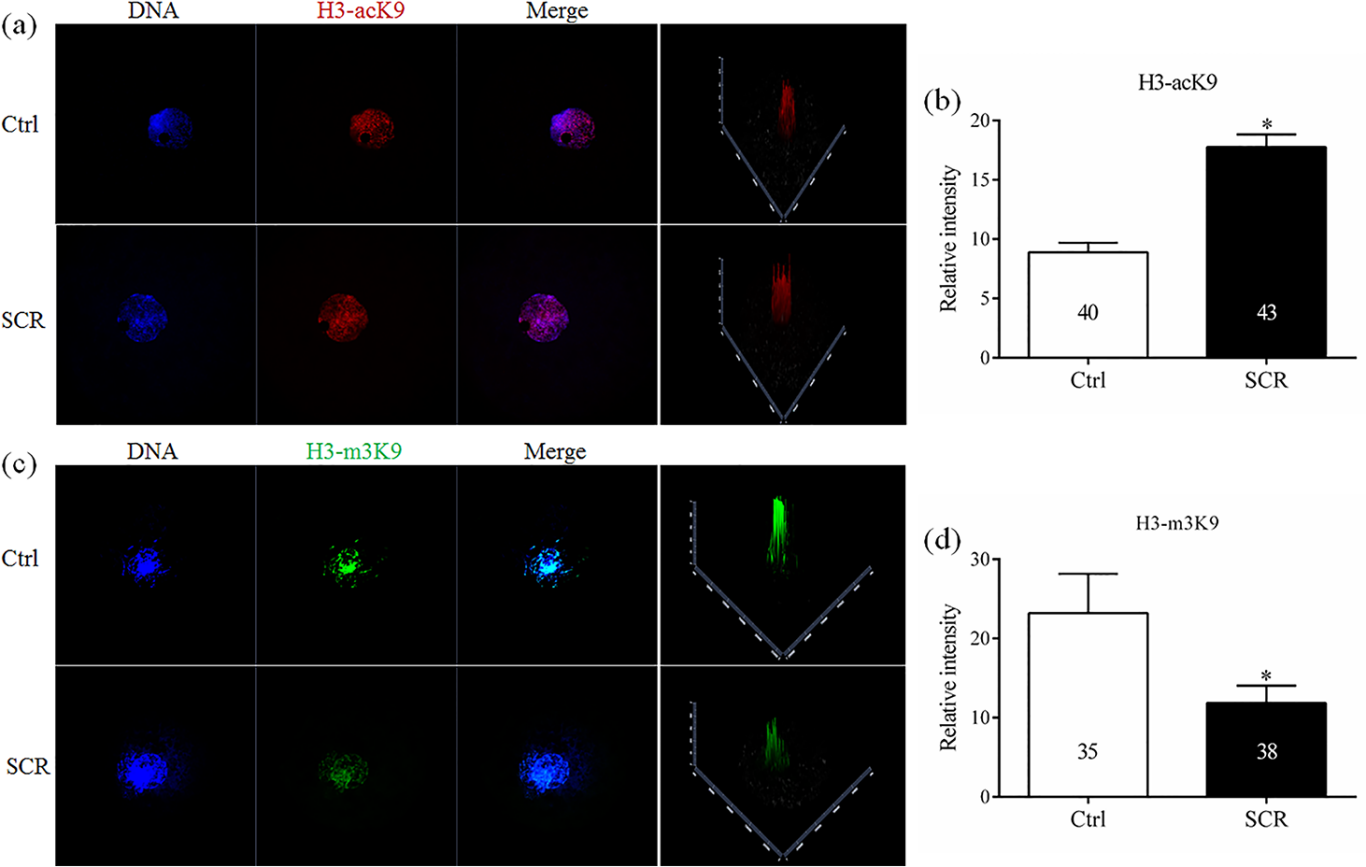
Laser scanning confocal microscopy images and quantification of levels of histone H3 acetylated at K9 (H3-acK9, a and b) and histone H3 trimethylated at K9 (H3-m3K9, c and d) in embryos at the pronuclear stage, with or without Scriptaid treatment. Embryos were labeled for H3-acK9 (red), H3-m3K9 (green), and DNA (Hoechst 33342, blue). The numbers of embryos examined in each experimental group are shown in the bars. * indicates P<0.05 compared with the control group. Values are the mean (± standard deviation of the mean) of four independent experiments. Scale bar, 20 μm. Ctrl: no treatment; SCR: Scriptaid treatment. Magnification, × 400.

**Fig 4:**
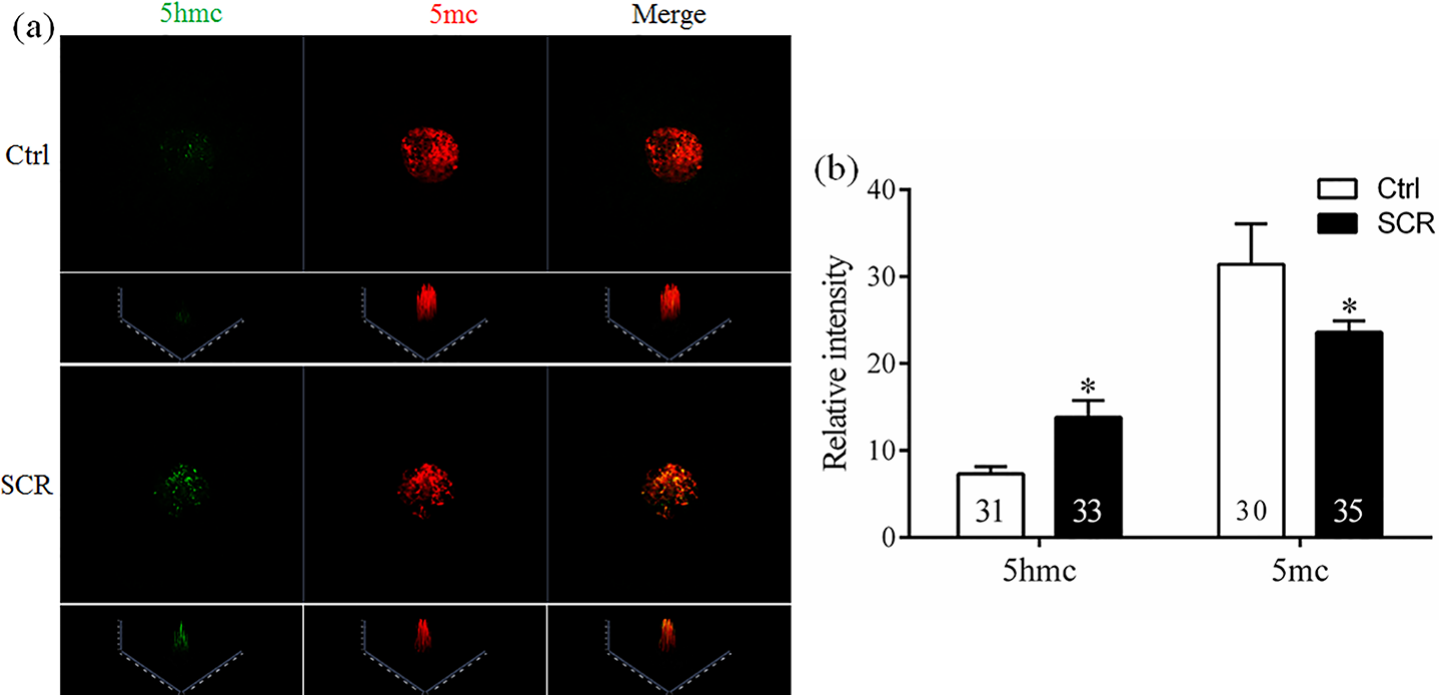
Laser scanning confocal microscopy images (a) and quantification (b) of the relative levels of 5-methylcytosine (5mc) and 5-hydroxymethylcytosine (5hmc) in early embryos at the pronuclear stage, with or without Scriptaid treatment. 5mc and 5hmc were detected using anti-5mc (red) and anti-5hmc (green) antibodies, respectively. * indicates P<0.05 compared with the control group. Values are the mean (± standard deviation of the mean) of four independent experiments. Ctrl: no treatment; SCR: Scriptaid treatment. Magnification, × 400.

**Fig 5:**
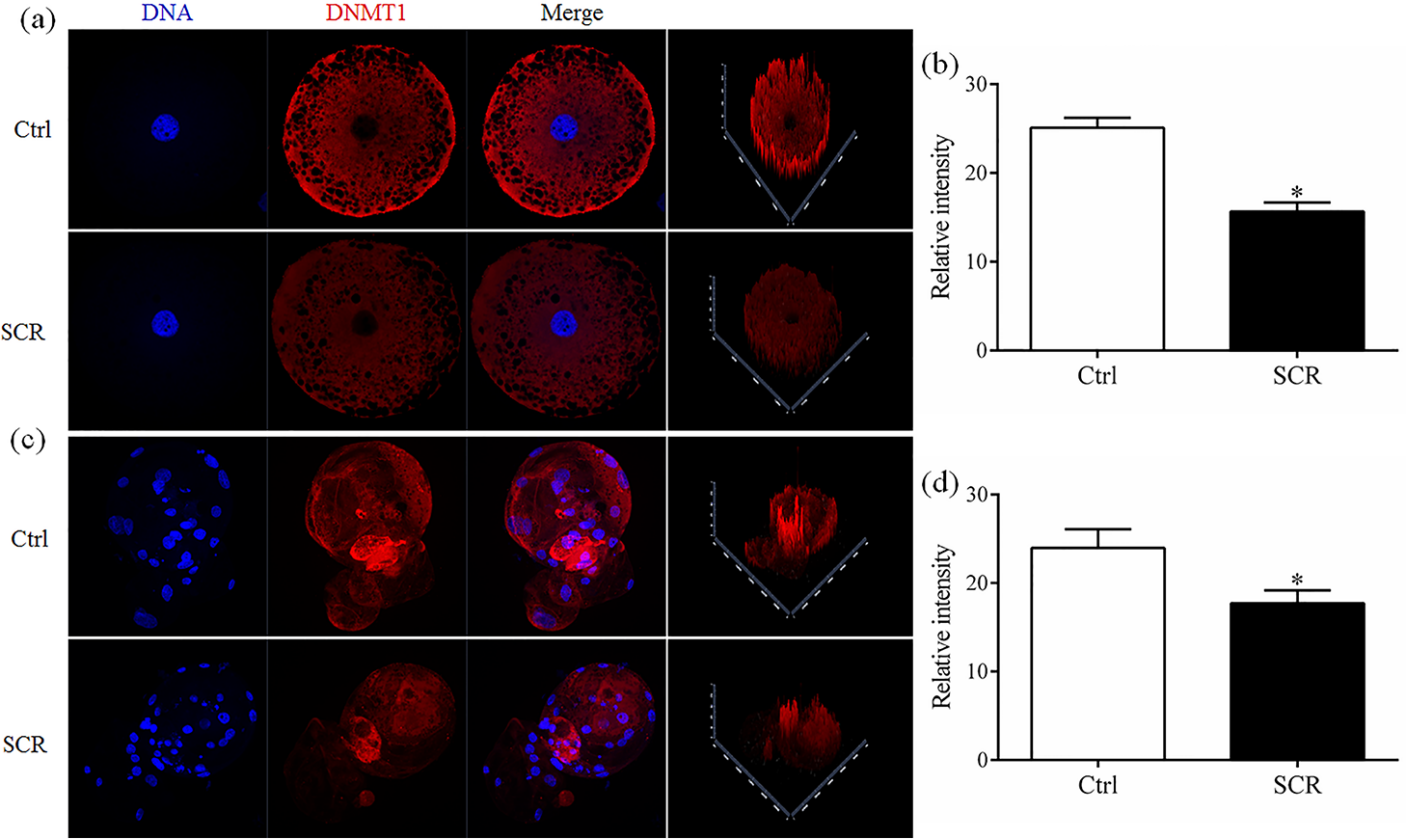
Laser scanning confocal microscopy images (a and b) and quantification (c and d) of the relative levels of *Dnmt1* in early embryos at the pronuclear and blastocyst stage, with or without Scriptaid treatment. The numbers of embryos examined in each experimental group are shown in the bars. * indicates P<0.05 compared with the control group. Values are the mean (± standard deviation of the mean) of four independent experiments. Ctrl: no treatment; SCR: Scriptaid treatment. Magnification, × 400.

**Fig 6:**
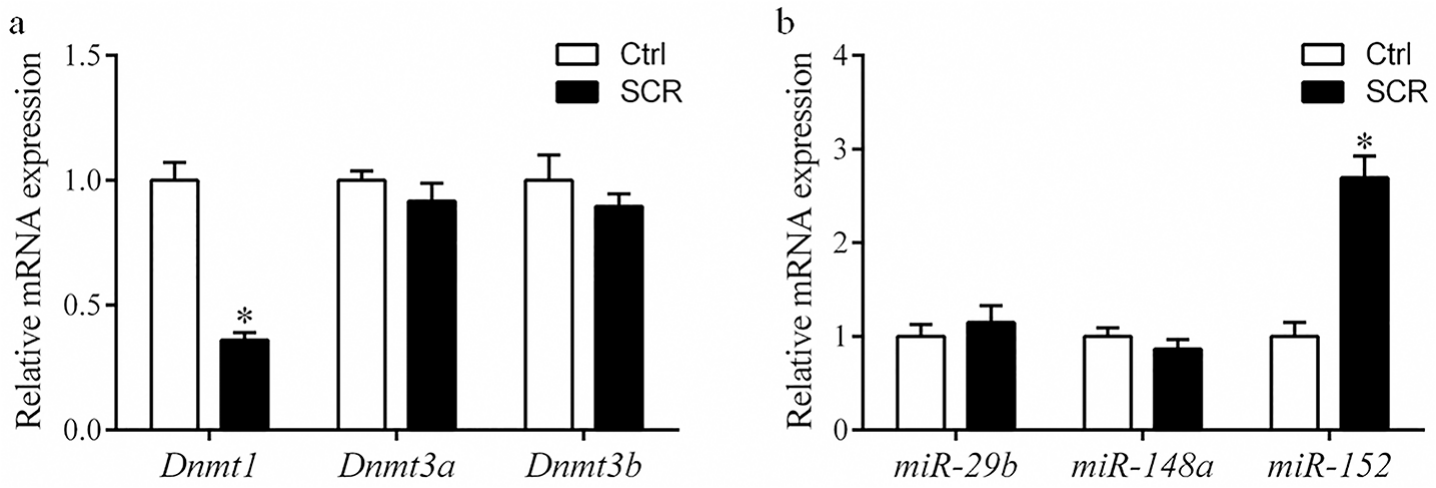
Relative mRNA levels of DNA methyltransferases (*Dnmt1*, *Dnmt3a*, and *Dnmt3b*; a) and microRNAs (*mir-29b*, *mir-148a*, and *mir-152*; b) in porcine blastocysts after 7 days of *in vitro* culture, with or without Scriptaid treatment. * indicates P<0.05 compared with the control group. Values are the mean (± standard deviation of the mean) of four independent experiments. Ctrl: no treatment; SCR: Scriptaid treatment. Magnification, × 400.

To analyze the mechanism underlying how Scriptaid treatment increases the developmental competence of SCNT embryos, levels of H3-acK9, H3-m3K9, 5mc, and 5hmc were examined in Scriptaid-treated SCNT embryos at the pronuclear stage. Scriptaid treatment significantly increased the level of H3-acK9 (Fig 3A and 3B) and significantly decreased the level of H3-m3K9 (Fig 3C and 3D) at the pronuclear stage. Meanwhile, conversion of 5mc into 5hmc was increased in Scriptaid-treated embryos (Fig 4A and 4B). Based on quantification of the fluorescence intensity of *Dnmt1* staining (Fig 5A and 5B), the level of *Dnmt1* at the pronuclear and blastocyst stage significantly decreased in Scriptaid-treated embryos. Furthermore, mRNA expression of *Dnmt1* was significantly lower in Scriptaid-treated embryos than in non-treated embryos (Fig 6A). To determine whether miRNAs are involved in downregulation of DNMT1, we compared the expression of miR-29b, miR-148a and miR-152 between Scriptaid-treated embryos and non-treated embryos. We found that the expression of miR-152, but not miR-29b and miR-148a, was significantly increased in Scriptaid-treated embryos, compared with non-treated embryos (Fig 6B).

### Effect of Scriptaid treatment on expression of POU5F1 and CDX2

**Fig 7:**
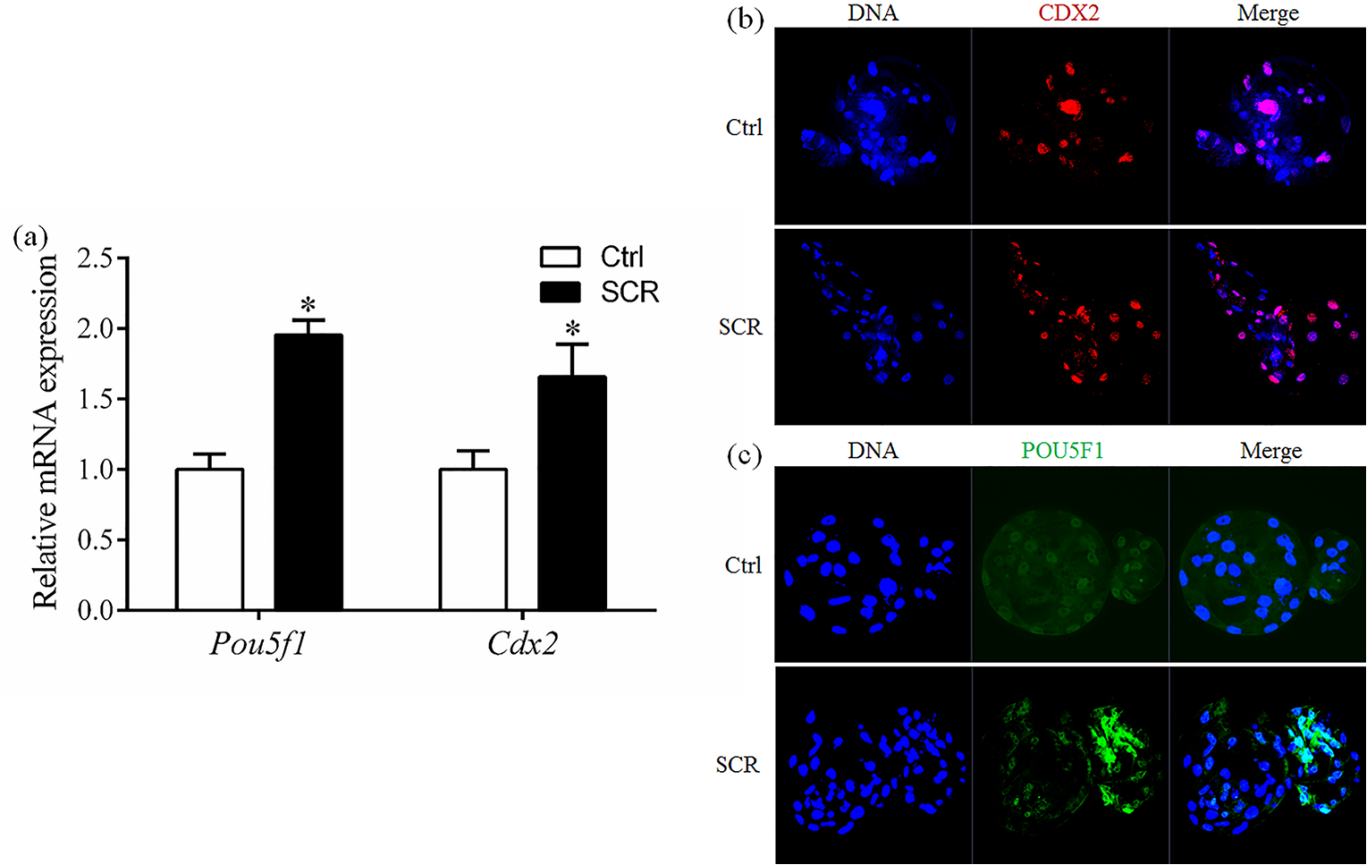
Relative mRNA expression levels (a) and laser scanning confocal microscopy images of immunostaining (b, c) for POU5F1 and CDX2 in porcine blastocysts after 7 days of in vitro culture, with or without Scriptaid treatment. * indicates P<0.05 compared with the control group. Values are the mean (± standard deviation of the mean) of four independent experiments. Ctrl: no treatment; SCR: Scriptaid treatment. Magnification, ×400.

mRNA and protein expression of two pluripotency-related genes, POU domain, class5, transcriptionfactor-1 (*Pou5f1*) and caudal type homeo box transcription factor 2 (*Cdx2*), was determined in SCNT embryos at the blastocyst stage. mRNA expression of *Pou5f1* and *Cdx2* was significantly higher in Scriptaid-treated embryos than in non-treated embryos (Fig 7A). Consistent with the mRNA results, protein expression of POU5F1 and CDX2 at the blastocyst stage was higher in Scriptaid-treated embryos than in non-treated embryos (Fig 7B).

## Discussion

Scriptaid is a novel HDACi that belongs to an existing class of hydroxamic acid-containing HDACis. In this study, we performed Scriptaid treatment after activation to improve epigenetic modifications in SCNT embryos and to increase their developmental competence.

Abnormal epigenetic reprogramming of donor nuclei after SCNT is thought to be the main cause of low cloning efficiencies. There are dynamic interactions between histone acetylation, histone methylation, and gene transcription [28–30]. Furthermore, previous studies have demonstrated that HDACi treatment could inhibit the histone deacetylase and results in hyperacetylation, which can expose DNA active binding sites and decrease DNA methylation that are important for gene activation [31]. K9 of histone H3 is an important site for both acetylation and methylation [32, 33]. In the present study, treatment with 300 nM Scriptaid for 20 h enhanced the level of H3-acK9 and reduced the level of H3-m3K9 in SCNT embryos at the pronuclear stage. Hypoacetylation of K9 of histone H3 is one of the major causes of the low cloning efficiencies of SCNT embryos in various species, including cattle [34], rabbits [35], and mice [23]. Our results suggest that Scriptaid treatment can significantly reduce the level of H3-m3K9 by increasing acetylation of K9 of histone H3. In addition, Scriptaid treatment increased conversion of 5mc into 5hmc.

In cloned embryos, the failure of DNA to be demethylated and remethylated causes incomplete nuclear reprogramming [36]. DNA methyltransferase *Dnmt1* is important for the maintenance of DNA methylation in mammalian cells [37, 38]. Accumulated evidence has been demonstrated that *Dnmt*s mediated transcriptional silencing in mammalian cells [39]. *Dnmt1* is also required to maintain patterns of DNA methylation and histone acetylation in cloned embryos; overexpression of *Dnmt1* causes abnormal embryonic development [40]. MicroRNAs (small non-coding RNAs, miRNA) play a critical role in maintenaning the pluripotent cell state and in the regulation of early mammalian development [39, 41]. Recent studies have been indicating that miRNAs involved into the regulation of pluripotency, not only in the reprogramming of embryos and somatic cells, but also participating the differentiation of stem cells [42, 43]. In our previous study, we showed that the expression levels of mir-127 and mir-136 between normal fertilized mouse embryos and cloned embryos are different [18]. In addition, deletion and knockdown of Xist, a non-coding RNA that control X-chromosome inactivation in female mammals, can increase cloning efficiency greatly, and correct epigenetic reprogramming errors [44]. These findings suggested that miRNAs played an important role during reprogramming of somatic cell. A recent study reported that miRNAs are involved in DNA methylation via their regulation of Dnmts [45]. mir-29a [46, 47], mir-148a, and mir-152 [48, 49] modulate Dnmt1 in cancer cells. In present study, we characterized the role of miR-152 in the regulation of DNA methylation in Scriptaid-treated porcine SCNT embryos for the first time. Our data showed that high level of miR-152 was associated with low Dnmt1 expression in Scriptaid-treated porcine SCNT embryos. Dnmt1 inhibition could be related with the overexpression of miR-152, meanwhile, resulting the conversion of 5mc into 5hmc to prevent hypermethylation. These findings indicated that miR-152 may have an indirect effect in DNA methylation. Therefore, we presume that Scriptaid treatment improves nuclear reprogramming in cloned embryos and its improvement of cloned embryo development might be owing to the correction of gene expression, including those encoding miRNAs.

Histone acetylases and deacetylases modulate the transcriptional activities of specific promoters by locally perturbing the chromatin structure, and increased histone acetylation can increase the transcriptional activities of genes [30]. In addition, methylation of H3K9 is causally linked to the formation of heterochromatin and long-term transcriptional repression [29]. Therefore, decreased methylation of K9 of histone H3 and DNA might improve the transcriptional activities of genes. To determine whether Scriptaid activates the expression of pivotal genes through histone acetylation in SCNT embryos, we examined the mRNA and protein expression of two genes that play important roles during development, namely, *Pou5f1* and *Cdx2*. Scriptaid treatment significantly increased the mRNA and protein levels of these two genes in porcine SCNT blastocysts, indicating that Scriptaid treatment can increase the transcriptional activities of genes in SCNT embryos. From our study, it could be implied that miR-152, which involved in the process of DNA demethylation of cloned embryos, will also initiate the expression of *Pou5f1* and *Cdx2*. Further supports the idea that Scriptaid treatment improves the developmental capacity and nuclear reprogramming of SCNT embryos.

Apoptosis occurs frequently during early embryonic development and has a marked impact on embryo development [50]. Therefore, we investigated the expression of three apoptosis-related genes, namely, *Bcl-xL*, *Bax*, and *Cas3*. *Bcl-xl*, which inhibits apoptosis, and *Cas3* and *Bax*, which promote apoptosis, belong to the *Bcl2* family. At least 11 *Caspase* genes have been identified that mediate protein cleavage and induce apoptosis. Among these, *Cas3* executes apoptosis. In this study, expression of *Cas3* and *Bax* at the blastocyst stage was lower in Scriptaid-treated embryos than in non-treated embryos. This indicates that Scriptaid treatment enhances the vitality of blastocysts. Moreover, expression of the apoptosis-inhibiting gene *Bcl-xL* at the blastocyst stage was higher in Scriptaid-treated embryos than in non-treated embryos. TUNEL assay also showed that Scriptaid inhibited apoptosis in blastocyst. These result demonstrated that Scriptaid treatment improves the quality of the produced blastocysts.

In conclusion, this study shows that DNMT1 and miR-152 expression induced by Scriptaid treatment enhances the development of reconstructed porcine embryos, modifies the epigenetic status, changes quality of blastocyst and level of apoptosis. Furthermore, *in vivo* produced embryos are suitable for use as control, as well as IVF embryos. However, there was extremely high possibility of polyspermic penetration during porcine IVF procedure [51, 52]. It is unlikely to get monospermic embryos in blastocyst stage. Thus according to the methods from previous studies [19, 21], non-treatment group was used as controls. We recommend treatment with 300 nM Scriptaid to improve the preimplantation development of porcine cloned embryos. Our findings demonstrate that this improvement is owing to enhanced epigenetic modification of somatic cells via Scriptaid-induced hyperacetylation and demethylation and upregulation of genes critical for SCNT embryonic development.
